# Preparation and Characterization of Alumina HDPE Composites

**DOI:** 10.3390/ma13010250

**Published:** 2020-01-06

**Authors:** Mohamed Saleh, Zainab Al-Hajri, Anton Popelka, Syed Javaid Zaidi

**Affiliations:** 1 Centre for Advanced Materials, Qatar University, Doha 2713, Qatar; misaleh@qu.edu.qa (M.S.); anton.popelka@qu.edu.qa (A.P.); 2 Department of Chemistry and Earth Science, Qatar University, Doha 2713, Qatar; zalahajri@qu.edu.qa

**Keywords:** high-density polyethylene (HDPE), porous alumina nanoparticles, thermal properties, morphology

## Abstract

In this study, effects of two different types of porous alumina nanoparticles have been incorporated into high-density polyethylene (HDPE) to study their impact on the properties of the HDPE composite. The dispersion of fillers in the HDPE matrix was evaluated by scanning electron microscopy (SEM). Differential scanning calorimetry (DSC) and thermogravimetric analyzer (TGA) integrated with Fourier transform infrared spectroscopy (FTIR) were applied to investigate the calorimetric behavior and thermal stability and to analyze the polymer decomposition, respectively. The dielectric properties were determined by a broadband dielectric spectroscopy. The effect of filler loading on the tensile properties and melt flow index was also examined. A homogenous distribution of the fillers was observed at low loading of alumina particles (below 5 wt. %). However, agglomerates of sub-micro size were formed extensively on samples with high loading of fillers (above 7 wt. %). A significant improvement of the thermo-oxidation stability of the composite was observed. The permittivity values of the prepared composites also increased with the addition of the fillers. The incorporation of fillers also increased the electrical conductivity values of the prepared composites at high frequencies.

## 1. Introduction

Polymer nanocomposites (PNCs) are a class of novel, advanced and high-performance materials which exhibit superior engineering properties (like barrier properties, permeation and flammability resistance) even at low loading level compared with conventional filler polymer blends. Therefore, these relatively new types of polymers have gained considerable attention in several industrial applications. PNCs are frequently prepared by adding a small number of nano-sized particles as reinforcement filler to the polymer matrix. However, the agglomeration and clustering of nanoparticles during PNC preparation have been a major challenge due to the mutual polar characteristics of many nanoparticles and non-polar polymers such as polyethylene (PE) and polypropylene (PP) [1,2]. The interfacial interaction between the nanoparticles and the polymer matrix plays an important role in achieving any improvement in the performance of PNCs [3].

Over the past few years, several methods have been developed to improve the dispensability of the nanoparticles in polyolefins. These methods include treatments of the nanoparticles using compatibilizers (e.g., silane coupling agents). However, the use of coupling agent is usually accompanied by higher costs. Furthermore, it is remarkable that many researchers succeeded in improving the physical and mechanical properties of PNCs without the aid of coupling agents [4,5,6]. Alumina nanoparticles can be considered as one of the most beneficial metal oxides with outstanding physical and chemical properties in analytical chemistry. Alumina (Al_2_O_3_) has several useful properties such as high insulation, good thermal stability, high strength and hardness, nontoxicity, good chemical resistivity as well as it’s low-cost compared to other metal oxide nanoparticles [7,8,9,10]. Therefore, the growing demand for developing new alumina PNCs for functional applications motivated scientists and engineers to conduct several studies in this research field [11].

Recently, studies of alumina PNCs have been conducted for many applications; Adhikari et al. (2006) investigated the effect of modified boehmite nanoparticles in polystyrene on the mechanical properties. A significant improvement in the mechanical performance was noticed at low loading levels of alumina nanoparticles (≈5 wt. %) with an aggregate size of less than 100 nm [12]. Khumalo et al. (2010) concluded that the addition of boehmite alumina to PE resulted in an enhancement of the thermo-oxidative degradation stability of the equivalent PE matrix [2]. Wang et al. (2015) studied the influence of alumina nanoparticles modified by vinyltrimethoxysilane on the improvement of dielectric properties, breakdown strength and volume resistivity of low-density polyethylene (LDPE) composites. The results showed the high suppressing effect of the modified alumina nanoparticles over the unmodified type. Also besides, it was observed that the modified alumina nanoparticles improved the breakdown strength and raised the volume resistivity compared with the pure polymer matrix [13].

Based on the previous findings of several studies, it can be concluded that the influence of nanofiller’s type and concentration on the properties of the obtained composites should be evaluated independently for every matrix and type of filler [14,15,16,17]. The present research aims to fabricate two types of alumina PNCs without neither performing any kind of surface treatment/modification nor using coupling agents. The effect of the nano-porous alumina particles on the morphology, thermal, mechanical and dielectric properties of HDPE composites was investigated. The loading of the fillers was varied between 0 and 10 wt. %.

## 2. Materials and Methods

### 2.1. Materials

**Figure 1:**
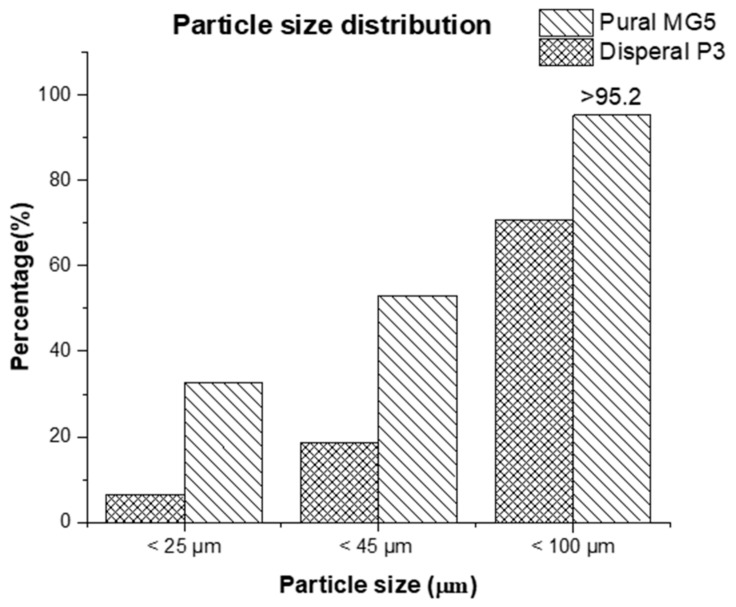
Particle size distribution of Disperal P3 and Pural MG5.

A pure HDPE (grade HHM 5202 BN) was provided by QChem Company LTD (Doha, Qatar). The nominal physical properties of pure HDPE are illustrated in Table 1. The alumina nanoparticles used in this study (Disperal P3 and Pural MG5) were kindly supplied by Sasol GmbH (Hamburg, Germany). A selection of certain physical properties of the fillers is listed in Table 2 and Figure 1.

### 2.2. Sample Preparation

The process of mixing the HDPE and the nanoparticles with different mass concentration (1, 3, 5, 7 and 10 wt. %) was carried out using a plastograph internal mixer Plastograph EC and Mixer 50EHT (Brabender, Germany) at 50 rpm for 10 min at 180 °C. The composites sheets (100 × 110 × 1.4 mm^3^) were prepared by a hot mounting press Carver 3895 (Carver, Wabash, IN, USA) at 180 °C with a holding time of 5 min and under a pressure of 2 MPa.

### 2.3. Surface Morphology Analysis

The morphology and dispersion of alumina particles were studied using SEM. A scanning electron microscope JCM 6000 (Jeol, Tokyo, Japan) at an accelerating voltage of 15 kV was used for these analyses. The samples for SEM examination were fractured after exposure to liquid nitrogen. The cross-sections of fractured samples were sputtered with a thin gold layer for better resolution.

### 2.4. Tensile Properties Investigation

The tensile strength and Young’s modulus were tested by a tensile testing machine LS 1K Plus (Lloyd Instruments, Largo, FL, USA) at a crosshead speed of 10 mm/min according to the ASTM D638-02 standard (Type V specimen) [18]. Five test replicates were performed for each blend and the average value was determined.

### 2.5. Melt Flow Index Measurement

The melt flow index (MFI) was tested using a melt flow indexer LMI 4000 (Qualitest, Lauderdale, FL, USA) and according to ASTM-D1238-13 at a temperature of 190 °C and under an applied load with a total mass of 2.16 kg [19].

### 2.6. Thermal Analyses

#### 2.6.1. DSC

The thermal properties were determined using a differential scanning calorimeter DSC 8500 (Perkin Elmer, Waltham, MA, USA). The tests were carried out at a heating rate of 10 °C/min under a constant flow of nitrogen between 30 °C up to 200 °C. The samples were heated at the beginning of the test from 30 °C to 200 °C to remove their prior thermal history, followed by cooling to room temperature. The heating cycle was repeated again to 200 °C.

The degree of crystallinity was calculated as per (Equation (1))

(1) $$Xc=\left(\Delta \mathrm{Hm}/\Delta {}^{\circ}\mathrm{Hm}\right)*100$$

where Xc is the degree of crystallinity; ΔHm is the melting enthalpy of the polymer, and the reference value (Δ°Hm) that was assumed in this study for 100% crystalline PE is 293 J/g [20].

#### 2.6.2. Thermogravimetric analyses (TGAs)

TGAs of the samples were performed using TGA 4000 (PerkinElmer, Waltham, MA, USA). TGAs were conducted in a nitrogen atmosphere in the temperature range of 30–600 °C at a heating rate of 10 °C/min.

### 2.7. Chemical Composition Investigation

FTIR was used for an investigation of the chemical composition of prepared composites. A Spectrum 400 (PerkinElmer, Waltham, MA, USA) spectrometer was used for obtaining FTIR spectra. The chemical composition of the pure HDPE, HDPE with Disperal P3 (HDPE-P3) and HDPE with Pural MG5 (HDPE-MG5) composites were investigated in a mid-infrared region (400–4000 cm^−1^).

### 2.8. Dielectric Properties

The frequency (f), electrical conductivity (Sig’) and relative permeability (ε) were measured by a broadband dielectric spectrometer BDS2400 (Novocontrol, Hundsangen, Germany). The samples (500 µm in thick, 20 mm in diameter) were placed between two gold-coated stainless-steel electrodes.

## 3. Results and Discussion

### 3.1. Morphology Investigation

**Figure 2:**
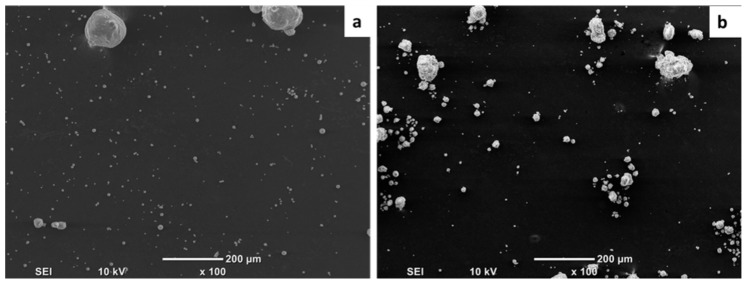
SEM micrographs of the fillers on a conductive adhesive tape: (**a**) P3; (**b**) MG5.

**Figure 3:**
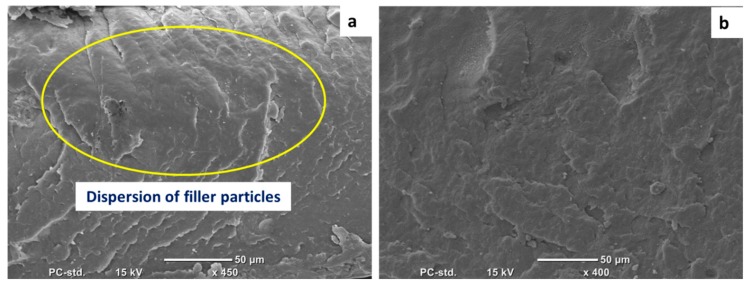
SEM micrographs of the cryo-fractured surfaces of (**a**) HDPE-1%P3 and (**b**) HDPE-1%MG5.

**Figure 4:**
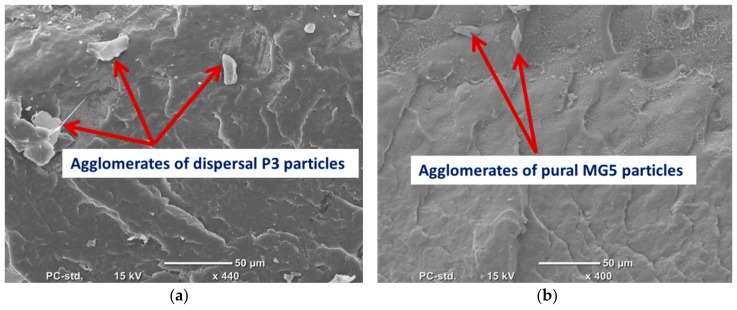
SEM micrographs of the cryo-fractured surfaces of (**a**) HDPE-5%P3 and (**b**) HDPE-5%MG5.

**Figure 5:**
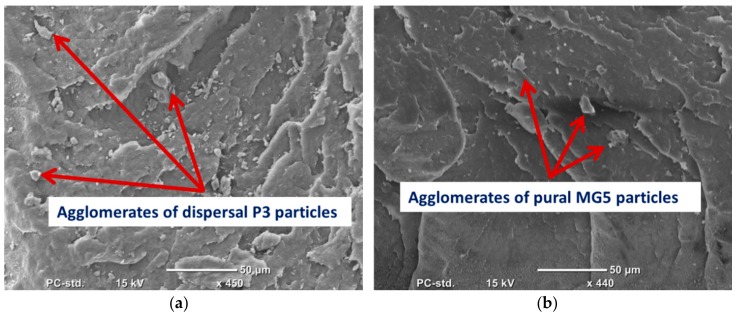
SEM micrographs of the cryo-fractured surfaces of (**a**) HDPE-10%P3 and (**b**) HDPE-10%MG5.

SEM was employed to evaluate the degree of filler dispersion in the polymer matrix. (Figure 2) shows the SEM micrograph of P3 and MG5 fillers dispersed on a conductive tape. It can be observed that the particle size of the fillers is on the nanoscale although some agglomerates were detected on the sub-micro scale, especially on the MG5 filler. The SEM morphologies taken from the cryo-fractured cross-sections of HDPE-P3 and HDPE-MG5 composites are presented in (Figure 3, Figure 4 and Figure 5). In most of the micrographs, the added fillers can be seen as small white spots. The fine dispersion of both fillers can be observed clearly, especially with a loading content of 1 and 3 wt. %. However, some agglomerates (indicated by red arrows in Figure 4 and Figure 5) with average sizes of 22–33 µm started to initiate at samples with 5 wt. % of the filler. It is worth noting that the rate of agglomeration in HDPE-MG5 composites is quite less than HDPE-P3 composites. In general, the SEM micrographs confirm that P3 and MG5 can be dispersed (up to 3 wt. %) in HDPE without undergoing any type of filler surface treatment and modification or adding polymeric coupling agents. However, the employment of certain surface functionalization on the alumina fillers may improve their degree of dispersion [13,20,21].

### 3.2. Mechanical Properties Analyses

**Figure 6:**
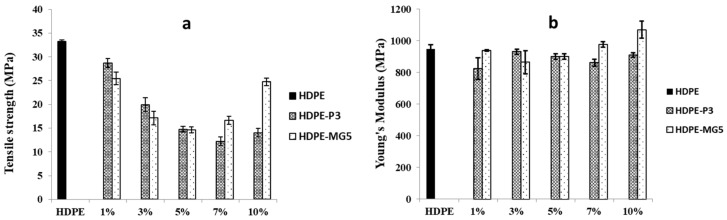
Mechanical properties of HDPE-P3 and HDPE-MG5 composites (**a**) tensile strength; (**b**) Young’s modulus.

The effect of alumina nanoparticles on the tensile strength and elastic modulus of the prepared HDPE composites is presented in Figure 6. The tensile strength decreased with the addition of P3 and MG5. However, the elastic modulus slightly increased with the incorporation of the alumina nanoparticles beyond 7 wt. % because the alumina nanoparticles inhibited the polymer matrix mobility. The values of tensile strength decreased due to the weak interaction between the filler and the polymer matrix, which was not strong enough to transfer the stress from HDPE to P3 and MG5 efficiently [22]. This finding is in line with the result reported by Sikora et al. (2019) [17]. It is noteworthy that the tensile strength of nanocomposites can be negatively affected by the filler content since the possibility of nanoparticles agglomeration increases with the higher filler concentration [12]. The particle size of the filler also plays an important role in improving the mechanical properties. Hamzah et al. (2015) noted a slight improvement in the tensile strength of polypropylene/ethylene propylene diene monomer rubber nanocomposites with the addition of alumina nanoparticles. However, the average particle size used in his study was only 12 nm. The particles with small size can interact with larger surface area in the system ensuring good adhesion between the filler and the polymer matrix [23,24].

### 3.3. Melt Flow Index Measurment

**Figure 7:**
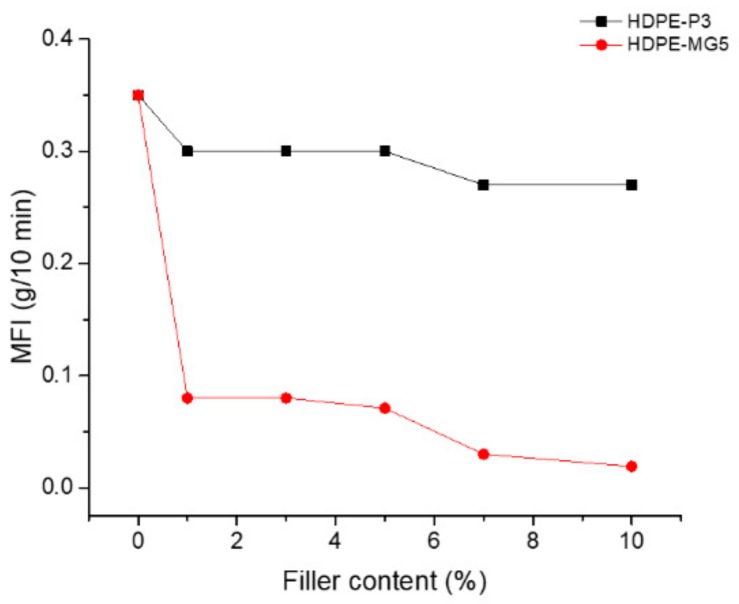
Effect of filler loading on the MFI of P3 and MG5 HDPE composites.

The effect of the two types of alumina nanoparticles on the melt flow index is given in Figure 7. As can be seen from Figure 7, the addition of both fillers caused a reduction in the MFI values, consequently, the viscosity of the prepared composites increased. However, the effect is more pronounced for the HDPE-MG5 than the HDPE-P3 sample. The MG5 alumina nanoparticles have low loose bulk density and higher surface area than the P3. Therefore, a limit on the content of fillers to be added to the polymer matrix should be considered. This reduction could be attributed to the interaction between the nanoparticles and the HDPE matrix, as well as the possibility of causing restrictions on polymer chain mobility due to the presence of the nanoparticles [22,25].

### 3.4. Thermal Properties Investigation

**Figure 8:**
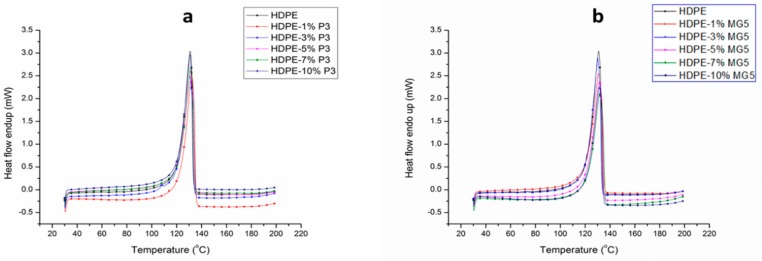
DSC curves from the second heating cycles of (**a**) HDPE-P3 composites and (**b**) HDPE-MG5 composites.

DSC tests were carried out to study the crystallization process, melting behavior and the induced influence on the mechanical properties of the prepared composites. Figure 8 shows the second heating cycle of HDPE-P3 and HDPE-MG5 composites. The peak shifting after adding the fillers was not significant. This means a negligible difference between the melting points of the pure HDPE and the prepared composites. Table 3 summarizes the melting and crystallization characteristics of the cooling and second heating cycles of HDPE-P3 and HDPE-MG5 composites. The heating cycles were characterized by two temperatures named as maximum melting point (T_m, max_) and final melting point (T_m, final_). However, the crystallization cycle was identified by maximum melting point (T_C, max_) and the initial crystallization point (T_C, initial_). The crystallinity percentage (Xc) was calculated by considering the ratio of the actual melting enthalpy of the composite to 100% crystalline PE (Δ°Hm = 293 J/g) [20]. The crystallization peak temperature of pure HDPE, which was obtained at 116 °C did not shift with the addition of P3 or MG5. The weak nucleation effect of both fillers is obvious and it is supported by both values of T_C, max_ and T_m, max_. The data obtained from the 2nd heating cycle shows that the incorporation of P3 and MG5 in the HDPE matrix resulted in a slight reduction in the crystallinity of HDPE composites. In addition, the melting behavior did not seem to be affected by the addition of the fillers. Similar behaviors have been reported by numerous studies, which examined the effect of alumina nanoparticles as nucleating agents. Khumalo et al. (2010) noted that the nucleating efficiency of boehmite alumina on the crystallization of HDPE and LDPE is low [2]. Mohamed et al. (2014) reported an approximate increase in the crystallization temperature of LDPE and linear low-density polyethylene (LLDPE) by 1–3 °C, upon the addition of different types of boehmite alumina [22].

**Figure 9:**
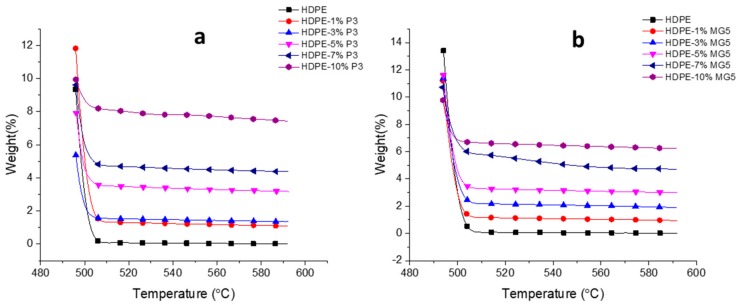
TGAs curves of (**a**) HDPE-P3 and (**b**) HDPE-MG5 composites.

Figure 9 and Table 4 show the TGAs curves and data (temperature at 5 wt. % weight loss and the residual amount of each blend at 592 °C in wt. %). A direct relationship between the thermal stability of the prepared PNC and its filler content can be clearly seen; the residual weight percentages of a sample noticeably increase with the increasing filler content. In addition, both type of fillers (MG5 and P3) almost show similar degradation profile. Similar results were reported by other researchers on different types of polymer matrices, like PE (HDPE and LDPE) and PP [2,21,26]. Generally, the improvement in the thermal stability could be attributed to the physical origin of the nanoparticles and due to their barrier effect towards diffusion of gaseous degradation products [27].

**Figure 10:**
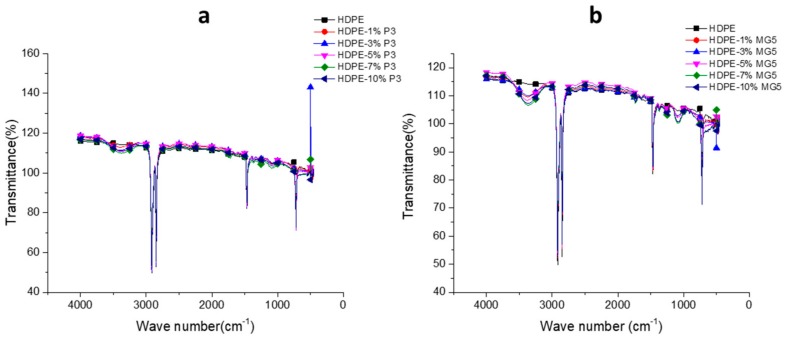
FTIR spectrums of (**a**) HDPE-P3 and (**b**) HDPE-MG5 composites.

The FTIR was performed to analyze the chemical composition of the prepared PNCs and to confirm chemical homogeneity of the fillers dispersed in the polymer matrices. Figure 10 shows the FTIR transmittance spectrum of neat HDPE and HDPE-P3 or HDPE-MG5 composites. The untreated HDPE spectrum was consisted of non-polar groups, such as bending in plane vibrations (asymmetric -CH_3_ at 1470 cm^−1^, asymmetric C-H at 1460 cm^−1^ and symmetric -CH_3_ at 1370 cm^−1^). The transmittance spectra peak that existed at the band region of 2857–2917 cm^−1^ is attributed to the rocking and stretching vibration of -CH_2_ bonds [28]. After mixing of HDPE with fillers, a transmittance peak can be clearly seen at 3500 cm^−1^ in the spectra of HDPE-P3 and HDPE-MG5 composites, which is attributed to the Al_2_O_3_ [13].

### 3.5. Dielectric Properties Analyses

**Figure 11:**
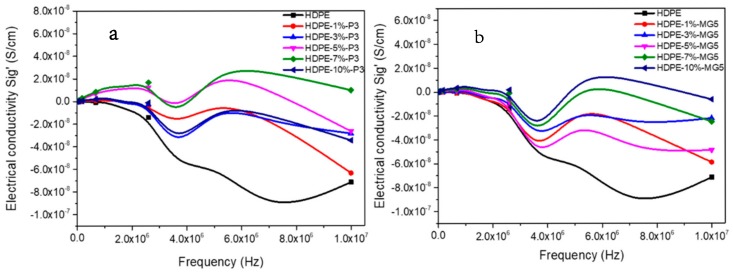
Electrical conductivity–frequency relationship of (**a**) HDPE-P3 and (**b**) HDPE-MG5 composites.

The electrical conductivity of HDPE-P3 and HDPE-MG5 composites with respect to the frequency is shown in Figure 11. In general, a direct relationship between the electrical conductivity with the percentage of the added filler can be noticed. Sig’ values tend to stabilize at low frequencies (at a frequency range of 0.01 to 1.89 Hz). The maximum Sig’ values of HDPE-P3 composites were recorded at a frequency range of 3.5 × 10^5^–1.3 × 10^6^ Hz followed by a significant reduction, while for HDPE-MG5 composites the Peak of Sig’ values was at the frequency range of 2.4 × 10^5^–9.4 × 10^5^ Hz.

**Figure 12:**
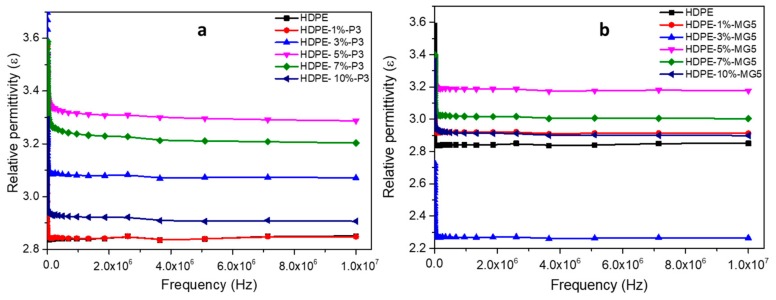
Relative permittivity–frequency relationship of (**a**) HDPE-P3 and (**b**) HDPE-MG5 composites.

**Figure 13:**
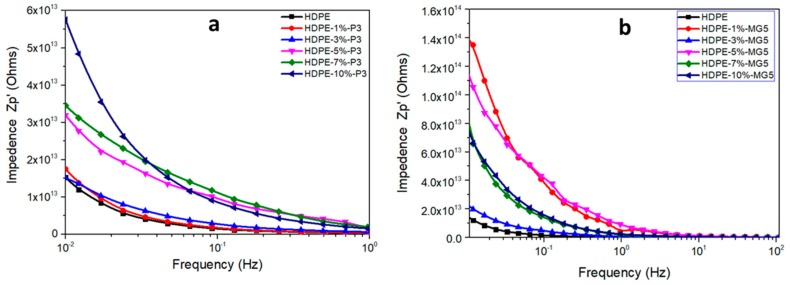
Electrical impedance–frequency relationship of (**a**) HDPE-P3 and (**b**) HDPE-MG5 composites.

Figure 12 shows the relative permittivity of HDPE-P3 and HDPE-MG5 composites as a function of frequency and within the range of 10^0^ to 10^7^ Hz at room temperature. The relative permittivity values (ε) increases with the decrease of frequency at the low-frequency region. This could be attributed to the electrode interfacial polarization, which occurs due to the difference in the conductivity and permittivity values of the fillers and the polymer matrix; ε values start to stabilize at a higher frequency range (over 10^3^ Hz) indicating the suitability of the usage of these composites in dielectric applications [13,29]. Wang et al. (2015) reported that the addition of alumina nanoparticles to LDPE inhibited the movement of charge carries, which resulted in a reduction in the relative dielectric permittivity [13]. A direct correlation also exists between the frequency and the electrical impedance (see Figure 13). Furthermore, the incorporation of alumina fillers increased the resistivity values.

## 4. Conclusions

In this study, the effect of two types of alumina nanoparticles (Disperal P3 and Pural MG5) on the morphology, tensile and thermal properties of the HDPE polymer was examined. The nanoparticles were incorporated from 1 up to 10 wt. % into the polymer matrix via melt compounding and hot press methods. The SEM micrographs showed that the fillers are finely distributed within the HDPE matrix. However, a direct correlation can be observed between the content of fillers and the tendency of agglomerates formation. The elastic modulus slightly increased with the incorporation of the alumina nanoparticles with a loading content above 7 wt. %. However, the values of tensile strength decreased due to the weak interaction between the filler and HDPE matrix. In addition, the viscosity of HDPE slightly increased with the addition of the fillers confirmed by MFI. Although the added nanoparticles showed a weak behavior as nucleating agents for crystallization, an improvement in the thermo-oxidation stability of the HDPE composites was recorded. The prepared HDPE-alumina composites can be used in dielectric applications; the stabilization of the relative permittivity values occurs at frequencies of 10^3^ Hz and beyond.

## Figures and Tables

**Table 1:** Nominal physical properties of high-density polyethylene (HDPE) HHM 5202 BN.

Physical Properties	Standard	Value
Density (g/cm^3^)	ASTM D1505	0.951
Melt Index [190 °C; 2.16 kg] (g/10 min)	ASTM D1238	0.35
Tensile yield strength [50 mm/min] (MPa)	ASTM D638	26
Elongation at break [50 mm/min] (%)	ASTM D638	>600
Brittleness temperature (°C)	ASTM D746	≤75

**Table 2:** Physical characteristics of dispersal P3 and Pural MG5.

Analysis	Disperal P3	Pural MG5
Surface area [550 °C/3 h] (m^2^/g)	317	355
Loose bulk density (g/mL)	0.92	0.52

**Table 3:** Melting and crystallization characteristics of HDPE and the prepared PNCs.

Sample	Crystallization Cycle			2nd Heating Cycle		
	T_C, max_ (°C)	T_C, initial_ (°C)	Xc (%)	T_m, max_ (°C)	T_m, final_ (°C)	Xc(%)
Pure HDPE	116.3	119.6	62.8	130.8	133.7	61.2
HDPE-1%P3	117	120.3	57	132	135.5	58.3
HDPE-3%P3	116.2	119.5	61.8	130.5	134.7	61.2
HDPE-5%P3	116.3	119.5	60.2	131	135	59
HDPE-7%P3	116.3	119.5	58.3	131.2	134.6	57.9
HDPE-10%P3	116.5	119.4	57.9	130.7	133.3	57
HDPE-1%MG5	116.1	119.1	63.1	130.7	135.2	61.5
HDPE-3%MG5	116.3	119.2	64.4	130	133.3	59.1
HDPE-5%MG5	116.2	119.6	60.7	131.3	134.7	57.7
HDPE-7%MG5	116.4	119.8	60.3	131.7	136	56.1
HDPE-10%MG5	116.2	119.6	58.5	131.5	134.6	55.5

**Table 4:** TGAs data of HDPE, P3 and MG5 composites.

Material	Temperature at Which—95 wt. % Loss (°C)	Residue at 592 °C (%)
HDPE	498	0.01
HDPE-1%P3	500	1.10
HDPE-3%P3	497	1.35
HDPE-5%P3	499	3.18
HDPE-7%P3	504	4.38
HDPE-10%P3	˃592	7.44
HDPE-1%MG5	498	0.95
HDPE-3%MG5	499	1.90
HDPE-5%MG5	499	2.99
